# Red imported fire ant nesting affects the structure of soil microbial community

**DOI:** 10.3389/fcimb.2023.1221996

**Published:** 2023-07-06

**Authors:** Jingjie Song, Zhenzhen Tang, Xueqing Zhao, Yanqiong Yin, Xiangyong Li, Fushou Chen, Aidong Chen, Ying Liu

**Affiliations:** ^1^ Key Laboratory of Green Prevention and Control of Agricultural Transboundary Pests of Yunnan Province, Agricultural Environment and Resources Institute, Yunnan Academy of Agricultural Science (YAAS), Kunming, China; ^2^ State Key Laboratory of Biocatalysis and Enzyme Engineering, School of Life Sciences, Hubei University, Wuhan, China

**Keywords:** RIFA, soil microbial community (SMC), 16S rDNA, invasion, ecosystem function

## Abstract

The red imported fire ants (RIFA, *Solenopsis invicta*) have become a well-known invasive species that poses significant ecological and economic threats globally. As of recent times, the geographic scope of its invasion in China is rapidly expanding, thereby aggravating the extent and severity of its detrimental effects. The importance of soil microorganisms for maintaining soil health and ecosystem function has been widely acknowledged. However, the negative impact of RIFAs on soil microbial communities and their functions has not yet been fully understood. In this study, we sequenced the V3-V4 variable region of the bacterial 16S rRNA gene in soil samples collected from three types of RIFA nests to investigate the impact of RIFA invasion on soil microbial diversity and composition. The results of alpha diversity analysis showed that the normal soil without nests of RIFAs exhibited the highest level of diversity, followed by the soil samples from RIFA-invaded nests and abandoned nests. Taxonomy and biological function annotation analyses revealed significant differences in microbial community structure and function among the different samples. Our findings demonstrate that RIFA invasion can significantly alter soil microbial community composition, which could ultimately affect ecosystem function. Therefore, effective management strategies are urgently needed to mitigate the negative impact of invasive species on native ecosystems.

## 1 Introduction

Red imported fire ant (RIFA, *Solenopsis invicta*) is one of the world’s most destructive invasive ant species which was listed as one of the world’s 100 worst invasive alien species by the International Union for Conservation of Nature (IUCN). RIFA was originated from South America, but now it has spread globally (Lowe et al., 2000). In China, RIFA invaded Taiwan in 2003 and was discovered in Guangdong in 2004, and had been listed as a plant quarantine pest since 2005 (Liu et al., 2021). RIFA has caused significant environmental, economic, and human health impacts in invaded areas (Vinson, 2013). In addition to its impact on biotic community, the negative impact of RIFA on the physical and chemical environment is profound. Their massive mounds, which can grow up to 40 cm in height, can cause soil erosion and destabilize infrastructure such as in roads and buildings (Poorter and Browne, 2005). Furthermore, the ants have been known to displace native ant species, thereby setting off an ecological cascade effect that affects plant diversity and nutrient cycling (Kannojia et al., 2019).

Soil microbiome plays a key role in regulating, supporting and supplying soil ecosystems. In the meanwhile, it is closely related to the cycling of soil organic matter, nitrogen and phosphorus, degradation of soil pollutants and rhizosphere immunity of soil-borne diseases (Chen S. C. et al., 2020; Dai et al., 2020; Wei et al., 2020). Soil microbial diversity is positively correlated with soil ecosystem versatility, and soil microbial communities with more diversity have the potential to improve soil ecosystem function. The functional redundancy caused by microbial diversity can improve the tolerance of ecosystem to stress (Xiong, 2022; Zhu et al., 2022). Loss of biodiversity and uniformity of soil community composition can impair and inhibit many ecosystem functions, including plant diversity, nutrient retention and nutrient uptake (Wagg et al., 2014). In the soil ecosystem, many microorganisms with different taxonomic positions can perform the same metabolic function. When a certain microorganism is expelled out by environmental stress, its role can be replaced by other species, so as to maintain the stability of the ecosystem, and similar phenomenon has been reported in other systems (Xu et al., 2019; Zhou et al., 2020). Therefore, the microbial diversity in soil is a driving force of the soil processes that are essential to sustaining agricultural production as a core soil health indicator (Kennedy and Stubbs, 2006). The soil microorganisms are a complex community, comprising bacteria, fungi, archaea, viruses, and protozoa that interact with each other and with plant growth promoting functions. Nutrient cycling is one of the critical functions of soil microorganisms, involving the breakdown of organic matter into simpler compounds that can be absorbed by plants. This process of mineralization includes several steps such as decomposition, ammonification, nitrification, and denitrification, during which microorganisms release enzymes that break down complex organic compounds, releasing nutrients like nitrogen, phosphorus, sulfur, and carbon into the soil. These nutrients support plant growth and development (Philippot et al., 2013; Chen Q. L. et al., 2020; Wilhelm et al., 2022). Thus, the structure and richness of soil microbial community are important indicators of soil health (Bardgett and van der Putten, 2014). Apart from nutrient cycling, soil microorganisms also contribute to soil structure and stability. Microorganisms produce substances that bind soil particles together, creating aggregates that improve soil porosity, water infiltration, and retention (Jacoby et al., 2017). These aggregates protect against soil erosion by reducing soil detachment and transport, thereby contributing to soil conservation. Additionally, soil microorganisms play a crucial role in suppressing plant pathogens and pests. Microorganisms compete with plant pathogens and pests for resources such as nutrients, space, and water, limiting their growth. Additionally, soil microorganisms can produce antimicrobial compounds that inhibit the growth and activity of plant pathogens and pests, including antibiotics (such as aminoglycosides, fluoroquinolones), hydrogen cyanide and siderophores. Thus, these microorganisms help to maintain health and productivity of plants, supporting both agricultural and ecological systems (Cycoń et al., 2019; Wilpiszeski et al., 2019; Abdul Rahman et al., 2021).

Recent research has also revealed that the soil microorganisms have a significant impact on plant growth and development through plant-microbe interactions. By forming symbiotic relationships with plant roots, microbes can enhance nutrient acquisition and improve plant resistance to biotic and abiotic stresses. In essence, soil microorganisms are a critical component of soil ecosystems that supports essential processes such as plant growth, nutrient cycling, soil structure formation, and pest suppression (Jacoby et al., 2017; Kannojia et al., 2019). As an invasive species with significant ecological and economic impacts caused by its rapid spread worldwide, although the impact of fire ant invasion on aboveground organisms has been extensively studied, the effects of these invasions on soil microorganisms have received less attention. In this study, we employed second-generation sequencing techniques to evaluate the impact of RIFA invasion on soil microorganisms by collecting soil samples from nesting, non-nesting and abandoned nest sites. Our analysis using next-generation sequencing revealed that RIFA invasion significantly impact soil microbial communities. The impact of RIFA invasion extends beyond aboveground organisms and has significant consequences on the health of the soil microorganism and the overall ecosystem functioning. It is crucial to understand these interactions to develop effective management strategies for invasive species and preserve the ecosystem’s health (Wetterer and Porter, 2003; King and Tschinkel, 2008).

## 2 Methods

### 2.1 Sample collection

Soil samples were collected from the abandoned land next to a park in Mengzi County, Yunnan Province, China, and covering an area of about 40 mu. The damage degree of RIFA reached level 4 or above, which belonged to the seriously harmful area. We collected SI_INS (*S. invicta* invaded nest soil sample) and SI_ANS (*S. invicta* abandoned nest soil sample) samples from active ant nests and abandoned ant nests over 15 centimeters above ground, and ensured that there were no other ant nests within 10 meters. Firstly, the above-ground portion of the nests and the soil 3 centimeters below the ground were removed at each study nests. Then, five 3 cm × 3 cm × 3 cm subplots were established within the range of the ant nest section, one at each direction and one in the center. The soil samples collected from the same nests at each plot were mixed into single composite samples, which were passed through 2 mm sieves and removed root materials and other impurities. The NS (normal soil sample) were taken from soil 3 to 5 cm below the ground where no other nests or ants were present, within 10 meters of ant nests. In total, 24 sieved soils (3 different samples, 8 replicates) were processed and stored as appropriate for further analyses.

### 2.2 DNA extraction

All the samples were selected to extract DNA of soil bacterial community. The total DNA from the samples was extracted with PowerSoil^®^ DNA Isolation Kit according to the manufacturer’s protocol. DNA quantity and quality were measured on a NanoDrop 2000 spectrophotometer (Thermo Fisher Scientific, USA). The integrity of the DNA after extraction was determined using a 1% agarose gel containing ethidium bromide. The extracted total DNA was preserved at −80 °C.

### 2.3 PCR amplification and high-throughput sequencing

The V3-V4 variable region of the bacterial 16S rRNA gene was amplified with primers 338F (5’-ACTCCTACGGGAGGCAGCA-3’) and 806R (5’-GGACTACHVGGGTWT CTAAT-3’). The 20 µL PCR reaction used TransGen AP221-02: TransStart Fastpfu DNA Polymerase. The 20 µL reaction system contained 4 µL of 5 × FastPfu Buffer, 2 µL of 2.5 mM dNTPs, 0.8 µL of 5 µM Bar-PCR primer F, 0.8 µL of 5 µM primer R, 0.4 µL of FastPfu polymerase, 0.2 µL of BSA, and 10 ng of genomic DNA. PCR amplification was conducted in an ABI GeneAmp^®^ 9700 thermocycler (IET, MA, USA) using the following protocol: 95 °C for 3 min followed by 30 cycles of 30 s at 94 °C, 30 s at 55 °C, and 45 s at 72 °C, and final extension for 10 min at 72 °C. The PCR products were examined by 2% agarose gel electrophoresis and purified using Agencourt AMPure XP beads (Beckman, Brea, CA, USA). Finally, the 16S rRNA gene amplicons quantified with 10 ng were pooled and subjected to the Illumina Novaseq6000 platform for sequencing at Beijing Tsingke Biotech Co., Ltd.

### 2.4 Statistical and bioinformatics analysis

The 16S rRNA gene sequences generated were pair-end merged, their chimeric sequences were removed. Using the software platform Usearch (version 7.0, http://drive5.com/uparse/, accessed on October 16, 2022) (Edgar, 2013), a higher-resolution equivalent of the operational taxonomic unit (OTU) at a 97% similarity level was obtained. Taxonomic analysis was then performed using Silva software for the bacterial 16S rRNA (http://www.arb-silva.de, accessed on October 16, 2022) (Pruesse et al., 2007; Pruesse et al., 2012; Klindworth et al., 2013; Quast et al., 2013; Beccati et al., 2017; Glöckner et al., 2017). To compare the values of alpha-diversity indices among the three soil groups, mean values were tested using R software (Version 4.1.2) with either a one-way ANOVA coupled with the Bonferroni test (equal variances) or the Dunnett’s T3 (unequal variances), or the nonparametric Kruskal-Wallis H test depending on the normality of the data. An array of alpha and beta diversity measures, including a principal coordinate analysis (PCoA) and non-metric multidimensional scaling (NMDS) on the OTU level based on the Bray-Curtis distance using the Vegan package in R(version 2.5.7) and ggplot2 (version 3.4.0). This was done to evaluate similarities between the samples. Within beta diversity (sample dispersion), an ANOSIM analysis based on Bray-Curtis was carried out using the R package Vegan (Version 3.4.0). The PICRUSt2 tool (https://github.com/picrust/picrust2, accessed on October 16, 2022) was also used to predict the functional category abundances for 16S rRNA gene sequence data by standardizing the OTU abundant table.

## 3 Results

### 3.1 Structural alterations of bacterial communities induced by the nesting behavior of RIFA

After quality filtering and removal of redundant sequences from 24 samples (NS, SI_INS, SI_ANS groups), a total of 2,159,764 high-quality sequences were retained in the 16S rRNA (V3-V4 region) sequencing database, with lengths ranging between 200 and 520 bp and an average length of 420 bp. Based on the 16S prokaryotic OTU identification pipeline, all detected OTUs belonged to the bacterial domain, and the 1357 OTUs were classified into 315 genera, 180 families, 103 orders, 40 classes, and 20 phyla.

The alpha diversity was estimated using the Sobs, ACE, Chao1, Shannon, and Simpson indices (**Table 1**). For the Sobs, ACE, and Chao1 indices, SI_INS exhibited the highest number of species, followed by NS, and then SI_ANS, which had the lowest number of species. This suggests that SI_INS has the greatest species richness, followed by NS and SI_ANS. However, for the Shannon and Simpson indices (which take into account both species abundance and evenness), NS had the highest diversity, followed by SI_INS, and SI_ANS. Species diversity is an important indicator of ecological health and stability. The results of the alpha diversity index analysis indicate that the invasion of red fire ants reduces soil microbial species diversity.

**Table 1:** Comparison of alpha diversity indices (Mean ± SD) among different groups.

Sample\Estimator	Sobs	Shannon	Simpson	Ace	Chao1
NS	411.13 ± 22.69^ab^	8.1432 ± 0.08979^a^	0.9951 ± 0.00033^a^	411.95 ± 22.73^ab^	417.84 ± 23.15^ab^
SI_INS	459.63 ± 9.98^a^	7.9969 ± 0.06413^a^	0.9921 ± 0.00027^b^	460.38 ± 9.95^a^	464.60 ± 9.75^a^
SI_ANS	406.63 ± 3.8^b^	7.7931 ± 0.014^ab^	0.9909 ± 0.00058^b^	407.30 ± 3.85^b^	411.09 ± 4.25^b^

Different letters indicate significant differences across treatments (one-way ANOVA, p < 0.05).

NS, normal soil sample; SI_INS, S. invicta invaded nest soil sample; SI_ANS, S. invicta abandoned nest soil sample.

**Figure 1:**
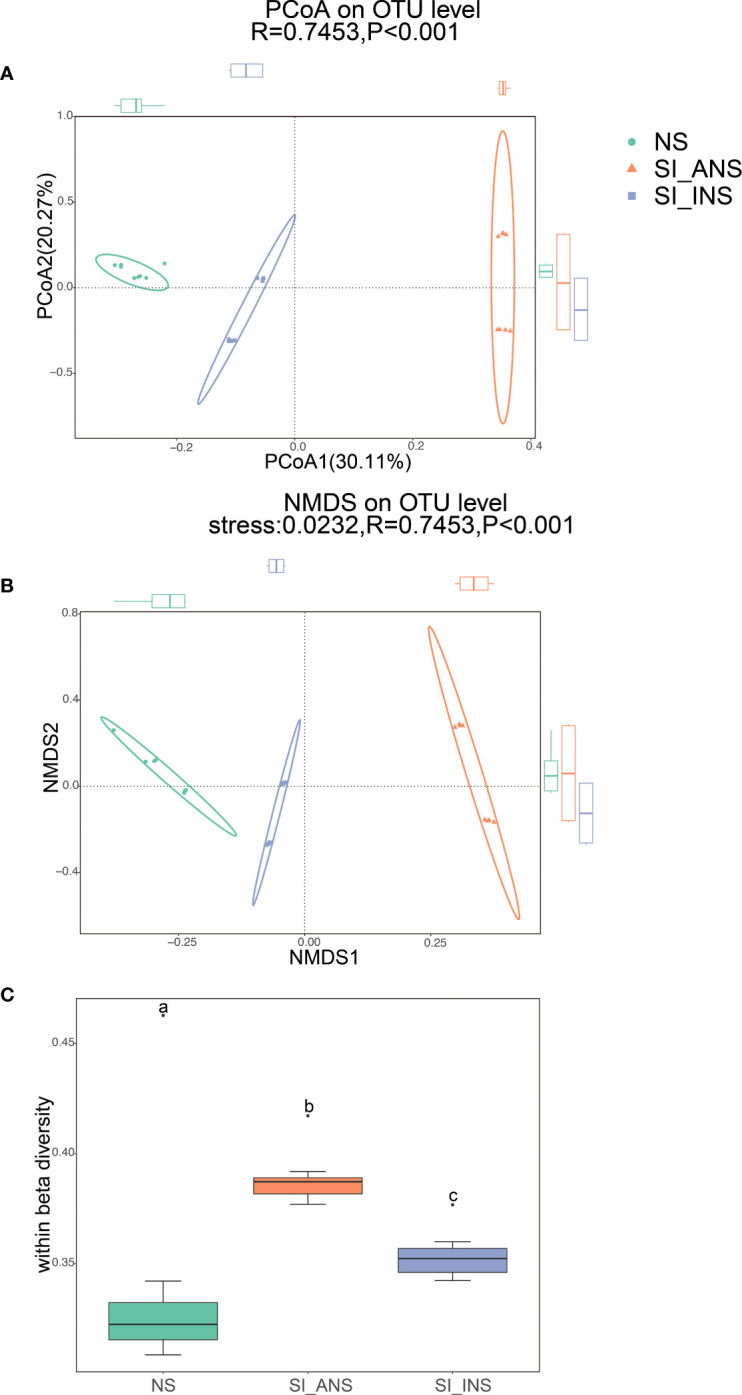
Beta diversity and within beta diversity analyses of three soil samples **(A)** PCoA (principal coordinates analysis) based on the Bray−Curtis distance at OTU level. **(B)** NMDS (non-metric multidimensional scaling) diagrams based on the Bray−Curtis matrix at OTU level. **(C)** At each group, variations of within beta diversity (sample dispersion) were measured as the distance of each sample from the centroid of each soil group. Distance was calculated using the OTU level of each sample group based on Bray−Curtis. The distribution of within beta diversity for each soil group was visualized using boxplot(reported data: minimum, first quartile, median, third quartile, and maximum). NS, normal soil sample; SI_INS, *S. invicta* invaded nest soil sample; SI_ANS, *S. invicta* abandoned nest soil sample. Different letters indicate significant differences across the treatments (one-way ANOVA, p < 0.05).

The results show that the three soil bacterial communities dispersed independently and distinctly from each other (**Figure 1A**; ANOSIM test, R = 0.7453, p < 0.001; **Figure 1B**; ANOSIM test, R = 0.7453, p < 0.001). PCoA and NMDS ordination also indicates a distinct microbiota for each soil group. Specifically, the scatter plot of the first two principal coordinates, PCO1 and PCO2, exhibits 30.11% and 20.27% of the data variation, respectively, and clearly separates each group. ANOSIM showed that the bacterial communities in each soil group formed distinct clusters, with NS having relatively stable bacterial communities that formed tight clusters in the ordination space. Regarding within beta diversity, NS demonstrated significantly lower variability than SI_INS and SI_ANS (**Figure 1C**; ANOSIM test, R = 0.6858, p < 0.001), which suggests that nesting behavior of Red imported fire ants significantly influenced sample-to-sample variability. Individuals in NS had the smallest dispersion, followed by those in SI_INS and SI_ANS.

### 3.2 Changes of bacteria community distribution and composition in soil induced by RIFA nesting

**Figure 2:**
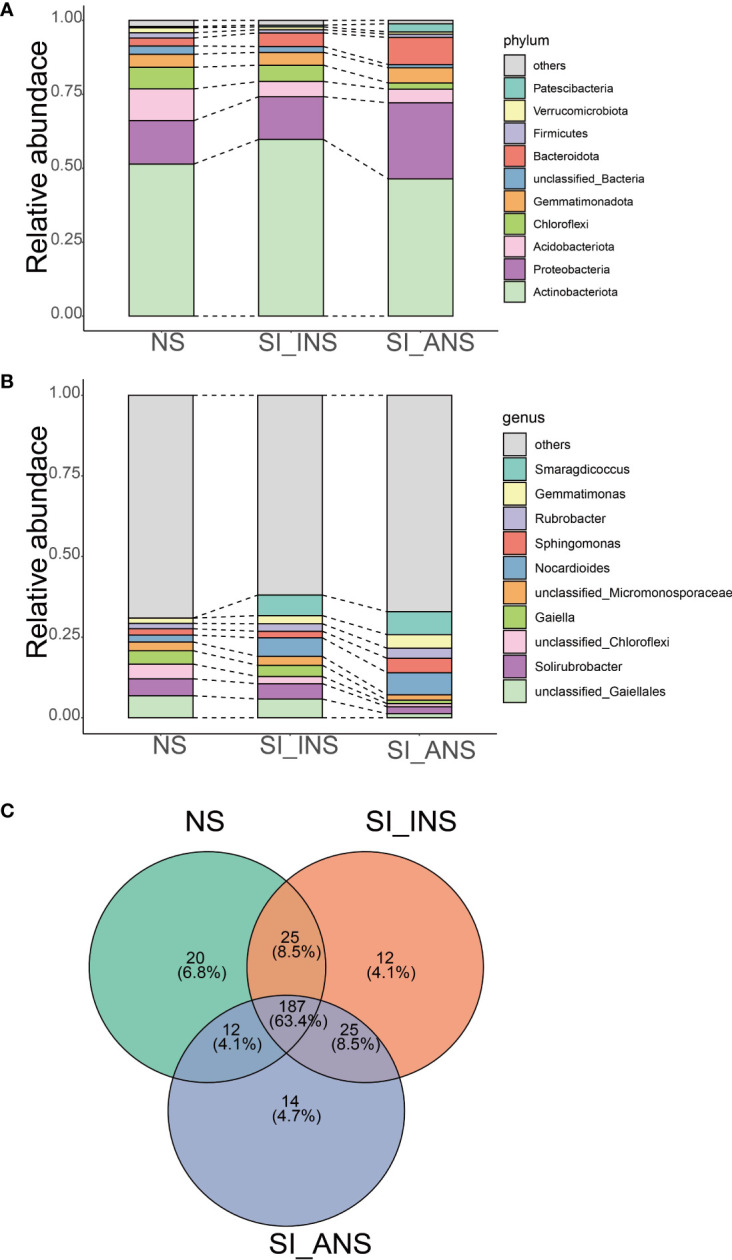
The relative abundance and composition of bacterial communities in different soil samples. **(A)** The relative abundance of bacteria communities at the phylum level. **(B)** Bacterial community relative abundance at the genus level. **(C)** Venn diagram of the shared and unique genera among different soil samples. The phyla and genera with a relative abundance not in top 10 were referred to as ‘others’. NS, normal soil sample; SI_INS, *S. invicta* invaded nest soil sample; SI_ANS, *S. invicta* abandoned nest soil sample.

The taxonomic analysis at the phylum level revealed that Actinobacteriota was dominant in NS (51.42%), SI_INS (59.75%) and SI_ANS (46.41%) (**Figure 2A**). The relative abundance of Actinobacteria was higher in SI_INS than in the other groups. We conducted analysis at the genus level for the 16S database, revealing the presence of bacteria associated with different soil samples (**Figure 2B**). The three different soil samples demonstrated distinct bacterial communities. NS was dominated by unclassified *Gaiellales* (6.82%), *Solibrubrobacter* (5.25%) and unclassified *Chloroflex*i (4.57%). In SI_INS, *Smaragdicoccus* was the most abundant genus (6.38%), followed by unclassified Gaiellales (5.80%) and Nocardioides (5.75%). The dominant genera in SI_ANS were *Smaragdicoccus* (7.13%), *Nocardioides* (6.84%) and *Sphingomonas* (4.46%). Across all soil samples, we identified a total of 187 genera that were shared among all three groups, mainly belonging to Actinobacteriota, Proteobacteria, Chloroflexi, Gemmatimonadota, Firmicutes and Patescibacteria (**Figure 2C**). NS had 20 unique genera, while SI_INS and SI_ANS had 12 and 14 unique genera, respectively (**Figure 2C**).

**Figure 3:**
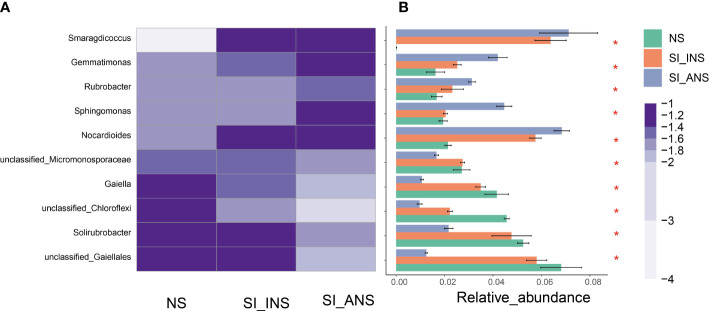
Comparison of the top 10 most abundant genera in three soil samples. **(A)** Heatmap of the relative abundance of the top ten shared genera. The colors indicate relative abundance ranging from grey (lower abundance) to purple (higher abundance). The color scale represents the normalized values of relative abundances by log10. **(B)** Relative abundances of the top ten genera that showed statistical differences among samples. NS, normalsoil sample; SI_INS, *S. invicta* invaded nest soil sample; SI_ANS, *S. invicta* abandoned nest soil sample; * means significance within the genus.

We present heatmaps that demonstrate the top 10 most abundant genera in each of the three soil samples (**Figure 3A**). The study found that *Nocardioides*, *Sphingomonas*, *Rubrobacter*, *Gemmatimonas*, and *Smaragdicoccus* displayed significantly higher levels of abundance in soil samples from RIFA-invaded nests compared to those from abandoned nests (Kruskal-Wallis H test, p < 0.05; **Figure 3B**). Moreover, unclassified *Gaiellales*, *Solirubrobacter*, unclassified *Chloroflexi*, *Gaiella*, and unclassified *Micromonosporaceae* were present in significantly higher amounts in normal soil sample and RIFA invaded nest soil sample, respectively, also compared to the RIFA abandoned nest soil sample (Kruskal-Wallis H test, p < 0.05; **Figure 3B**). The results also exhibit a decreased abundance of *Gaiella* and unclassified *Gaiellales*, while *Nocatdioides*, *Gemmatimonas*, and *Smaragdicoccus* increased significantly in RIFA invaded nest soil. These findings provide insights into the differences in bacterial community composition among the three soil samples analyzed in this study. The data suggest that the abandoned RIFA nest (SI_ANS) may support a distinct bacterial community compared to the other two soil samples.

### 3.3 Function profiling of soil bacterial community before and after the invasion of RIFA

**Figure 4:**
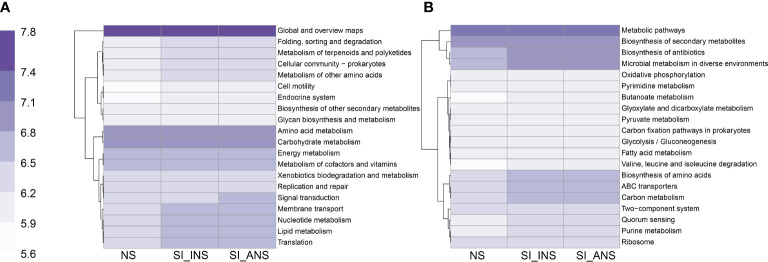
Prediction of KEGG functions for three different soil samples. **(A)** Function prediction in pathway level two. **(B)** Function prediction in pathway level three. NS, normal soil sample; SI_INS, *S. invicta* invaded nest soil sample; SI_ANS, *S. invicta* abandoned nest soil sample.

PICRUSt analyses revealed that folding sorting and degradation, metabolism of terpenoids and polyketides, cellular community, metabolism of other amino acids, membrane transport, nucleotide metabolism, lipid metabolism, and translation were all enriched in both SI_INS and SI_ANS at the pathway level two compared to NS samples (**Figure 4A**). Additionally, at the pathway level three, biosynthesis of antibiotics, microbial metabolism in diverse environments, butanoate metabolism, glyoxylate and dicarboxylate metabolism, pyruvate metabolism, carbon fixation pathways in prokaryotes, glycolysis/gluconeogenesis, fatty acid metabolism, valine, leucine and isoleucine degradation, biosynthesis of amino acids, ABC transporters, two-component system, quorum sensing, and purine metabolism were enriched in SI_INS and SI_ANS (**Figure 4B**).

## 4 Discussion

To explore the impact of red imported fire ants on soil microbial communities, we assessed both alpha-diversity and beta-diversity metrics. Our analysis revealed that the Chao1 index and Shannon index were significantly higher in SI_ANS than in NS and SI_INS, indicating a more diversified community structure in the abandoned nest soils. This could be attributed to the fact that the abandoned nests provided a favorable environment for a more diverse microbial community to develop. Regarding beta-diversity, we conducted PCoA analysis based on Bray-Curtis dissimilarity indices to investigate the differences in microbial community composition among the three groups. Our results showed that the microbial communities in SI_ANS were clearly separated from those in NS and SI_INS, indicating a significant difference in community composition between the abandoned nest soil and the soil samples collected from red imported fire ant-infested areas. Furthermore, the PERMANOVA test indicated that there was a significant difference in microbial community composition among the three groups (p < 0.05). These findings suggest that the invasion of red imported fire ants may alter soil microbial community composition and facilitate the establishment of a unique microbial community structure.

Our analysis revealed the enrichment of certain microbial species in each soil sample. For instance, we observed a significant increase in the abundance of Gaiellales in NS and SI_INS compared to SI_ANS. Previous studies indicate that Gaiellales have the potential to treat sewage and waste by converting them into valuable biomass (Severino et al., 2019). These findings suggest that the higher abundance of Gaiellales in the soil samples of NS and SI_INS could have a potential application in soil improvement and plant growth. In contrast, the significant decrease in the abundance of Gaiellales in the nest soil abandoned by red fire ants indicates a decline in the carbon cycle and soil amelioration ability of the soil microbial community. We also found that *Solibacter* strains were generally more abundant in the soil samples of NS and SI_INS than in SI_ANS. *Solibacter* are widely distributed in natural environments and are known for their extreme tolerance to various conditions (Eichorst et al., 2018; Lopez-Fernandez et al., 2021). They play vital roles in biogeochemical cycling, such as the decomposition of organic matter, carbon fixation, and nitrogen cycling, which makes their abundance an indicator of soil health (Cong et al., 2015; Buscardo et al., 2018). However, the higher abundance of *Solibacter* in NS and SI_INS compared to SI_ANS may also indicate a negative impact of red imported fire ants on soil quality.

The abundance of *Nocardioides* bacteria was significantly higher in SI_INS and SI_ANS than in NS. Some studies suggested that *Nocardioides* strains may release volatile compounds, such as geosmin and 2-methylisoborneol, which may attract red fire ants (Huang et al., 2020). Additionally, studies have found that *Nocardioides* bacteria may have antibiotic-producing ability, which can affect the growth and development of other microorganisms (Amaning Danquah et al., 2022). This could make the area more suitable for the survival and reproduction of red fire ants. On the other hand, the abundance of *Sphingomonas* bacteria was higher in SI_ANS than in NS and SI_INS. Studies suggest that *Sphingomonas* bacteria can degrade geosmin, a volatile compound with a strong fishy odor, which could explain why the abundance of *Sphingomonas* and *Nocardioides* bacteria in SI_ANS is quite high in the abandoned ant nest soil (Clercin et al., 2021). It was found that *Smaragdicoccus* belongs to a group of bacteria that utilize natural substances and requires a large amount of organic matter such as fibers and plant residues for its growth and reproduction (Ma et al., 2022). In normal soil, this kind of organic matter is relatively low, which makes it difficult for *Smaragdicoccus* to occupy a high abundance. However, the nest soil of red ants contains a large amount of fibers and plant residues, which may have led to a higher abundance of *Smaragdicoccus* in SI_INS, SI_ANS compared to NS.

Moreover, PICRUSt results revealed that several KEGG pathways were enriched in SI_INS and SI_ANS, including pathways related to metabolism, membrane transport, and quorum sensing. These results highlight the functional similarities between SI_INS and SI_ANS, which differ substantially from NS. Our findings provide important information for understanding how soil microbial communities respond to changes in environmental conditions and can be used to manage soil fertility and health. Overall, our study highlights the importance of considering both taxonomic and functional characteristics of microbial communities when studying soil ecology. The insights gained from our analyses have the potential to inform future studies investigating the role of microbial communities in soil and their response to environmental changes. These findings suggest that the invasion of RIFA alters the functional potential of the soil microbiota, affecting various soil processes such as nutrient cycling, decomposition, and organic matter turnover. The enrichment of antibiotic biosynthesis pathways in SI_INS and SI_ANS could potentially increase antibiotic resistance among soil bacteria, having ecological and public health implications. However, we must note that the resolution of PICRUSt remains low and may contain some biases, requiring further bioinformatics or experimental evidence to verify (Visioli et al., 2018).

In conclusion, our study demonstrated that the invasion of red imported fire ants significantly impacted not only alpha-diversity and beta-diversity metrics but also functional potential of soil microbial communities. By providing new insights into the complex interactions between red imported fire ants and soil microbial communities, our findings underscored the importance of effective management strategies to mitigate the negative impact of these invasive species on native ecosystems. Future research should focus on understanding the mechanisms underlying the observed changes in microbial communities and the potential consequences for soil processes and ecosystem functioning.

## Data availability statement

The data presented in the study are deposited in the PRJNA973254 repository, accession number SRP438159.

## Author contributions

Concepts: YL and AC; Design: YL, JS, and ZT; Experimental studies: JS, ZT, XZ, YY, XL, and FC; Data acquisition: JS, ZT, XZ, YY, XL, and FC; Data analysis: JS and ZT; Statistical analysis: YL, JS, and ZT; Manuscript preparation: JS and ZT; Manuscript editing: YL, JS, ZT, and AC; Manuscript review: YL and AC. All authors contributed to the article and approved the submitted version.
